# Carbenoid-mediated nucleophilic “hydrolysis” of 2-(dichloromethylidene)-1,1,3,3-tetramethylindane with DMSO participation, affording access to one-sidedly overcrowded ketone and bromoalkene descendants^§^

**DOI:** 10.3762/bjoc.10.28

**Published:** 2014-01-31

**Authors:** Rudolf Knorr, Thomas Menke, Johannes Freudenreich, Claudio Pires

**Affiliations:** 1 Department Chemie, Ludwig-Maximilians-Universität München, Butenandtstrasse 5–13 (Haus F), 81377 München, Germany

**Keywords:** brominative deoxygenation, carbenoid, DMSO, nucleophilic vinylic substitution, steric hindrance

## Abstract

2-(Dichloromethylidene)-1,1,3,3-tetramethylindane was “hydrolyzed” by solid KOH in DMSO as the solvent at ≥100 °C through an initial chlorine particle transfer to give a Cl,K-carbenoid. This short-lived intermediate disclosed its occurrence through a reversible proton transfer which competed with an oxygen transfer from DMSO that created dimethyl sulfide. The presumably resultant transitory ketene incorporated KOH to afford the potassium salt of 1,1,3,3-tetramethylindan-2-carboxylic acid (the product of a formal hydrolysis). The lithium salt of this key acid is able to acylate aryllithium compounds, furnishing one-sidedly overcrowded ketones along with the corresponding tertiary alcohols. The latter side-products (ca. 10%) were formed against a substantially increasing repulsive resistance, as testified through the diminished rotational mobility of their aryl groups. As a less troublesome further side-product, the dianion of the above key acid was recognized through carboxylation which afforded 1,1,3,3-tetramethylindan-2,2-dicarboxylic acid. Brominative deoxygenation of the ketones furnished two one-sidedly overcrowded bromoalkenes. Some presently relevant properties of the above Cl,K-carbenoid are provided in Supporting Information File 1.

## Introduction

**Scheme 1:**
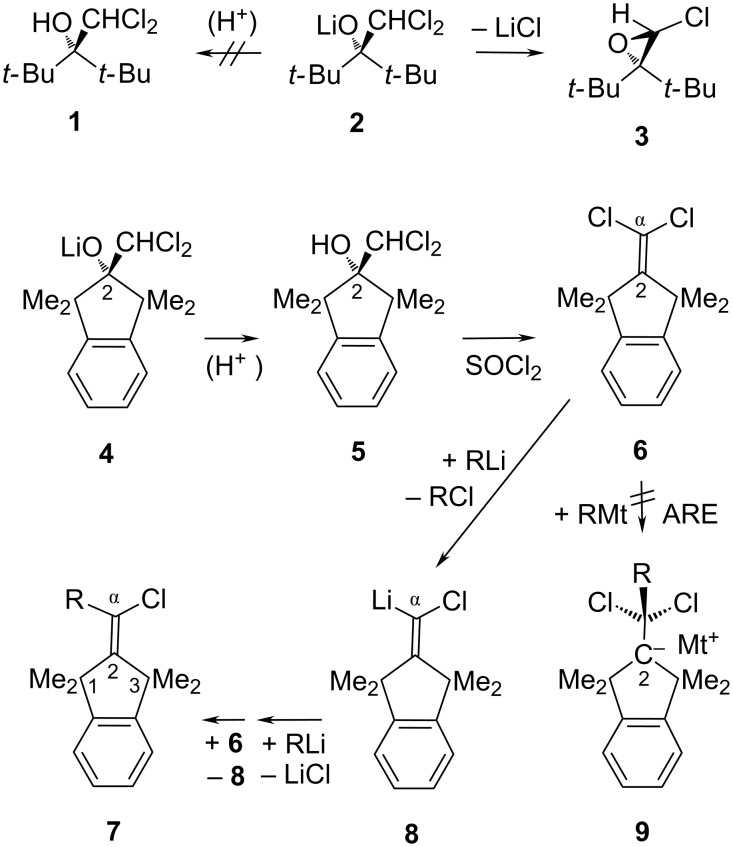
1,1,3,3-Tetramethylindane derivatives are preferable.

The 1,1,3,3-tetramethylindan-2-yl(idene) fragments shown in the hydrocarbon parts of formulae **4**–**8** (Scheme 1) are preferable to the corresponding acyclic di-*tert*-butylmethylidene moiety (*t-*Bu_2_C in **1**–**3**) as the shielding substituent in static and dynamic model systems for several reasons. (i) With respect to repulsive strain, an attempted protonation of the alkoxide **2** immediately after its generation [2] at −70 °C failed to provide **1**, because **2** cyclized too rapidly with formation of the chlorooxirane **3**. On the other hand, the somewhat alleviated internal repulsion in alkoxide **4** allowed it to be trapped by protonation below −10 °C before the cyclization could interfere [3], so that the resultant alcohol **5** could be isolated (crude yield 90% from 1,1,3,3-tetramethylindan-2-one) and dehydrated to give 2-(dichloromethylidene)-1,1,3,3-tetramethylindane (**6**) as the only product (97% yield). (ii) In X-ray diffraction analyses [4–9], the 1,1,3,3-tetramethylindan-2-ylidene parts turned out to be rather rigid, except for an occasional folding along the C-1/C-3 axis, and they did not exhibit the structural disorder problems and vexing angular flexibility which can arise with the *t-*Bu_2_C groups exemplified in Scheme 1. (iii) Depending on the substituents at the exocyclic C-α atom in **7**, all four methyl groups in the 1,1,3,3-tetramethylindan-2-ylidene parts [10] and in their truncated analogue 1,1,3,3-tetramethylcyclopent-2-ylidene [11] may be nonequivalent and provide useful stereochemical and stereodynamic NMR information that would not be available from models containing the *t-*Bu_2_C moiety with free rotation about the *t-*Bu–C bonds. (iv) Vinylic nucleophilic substitution (S_N_V) of the chloride anion from **6** by even a very strong nucleophile R–Mt (Mt = alkali metal) to give **7** may appear problematic, because the first intermediate **9** expected with the usual ARE (addition–rotation–elimination) [12] mechanism would suffer from poor stabilization of the negative charge at C-2 which is flanked by two *tert*-alkyl groups. Instead, the substitution products **7** were obtained from **6** in THF via the cyclic Li,Cl-carbenoid **8** at room temperature (rt) by the carbenoid chain mechanism [3], as indicated in the bottom line of Scheme 1. These S_N_V reactions proceed properly because **8** has a reduced (albeit not vanishing) inclination toward cycloalkyne formation through the Fritsch–Buttenberg–Wiechell (FBW) rearrangement [13], whereas S_N_V reactions of acyclic Mt,Hal-carbenoids often have to compete with FBW processes forming acyclic alkynes.

The α,α-dibromo analogue of **6** (available [14] from **6** in two steps) was found to undergo corresponding but more intricate carbenoid chain reactions. Therefore, it was planned to prepare α-bromo analogues of **7** from related ketones which should be accessible via the carboxylic acid **10** to be expected from a hydrolysis of **6**.

## Results and Discussion

### “Hydrolysis” of the α,α-dichloroalkene **6**

**Scheme 2:**
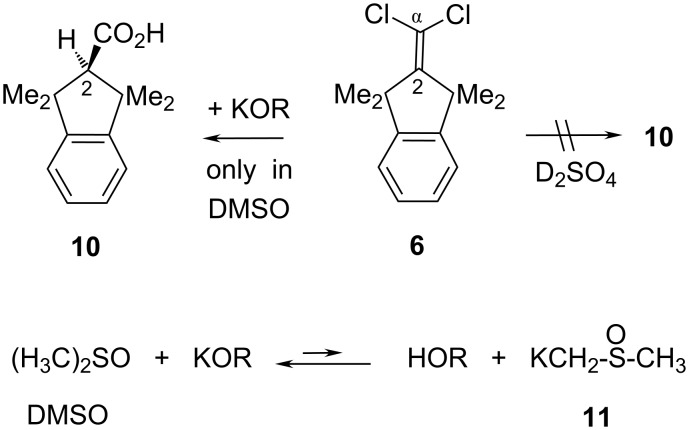
“Hydrolysis” of **6**, and the KOR/dimsyl-K (**11**) system.

The carboxylic acid **10** (Scheme 2) would normally (and more expediently than before [15]) be accessible through a simple hydrolysis reaction [16–17] of **6** with concentrated (80–100%) sulfuric acid. Our in situ ^1^H NMR spectra showed that **6** was insoluble in D_2_SO_4_ (97%) at rt and that two promising, equally intense methyl singlet signals, as expected for **10**, appeared after seven hours at 100 °C. However, the usual (Et_2_O/water) work-up procedure did not afford any (acidic or nonacidic) organic product, which suggests that **6** or **10** were converted to water-soluble, unserviceable arenesulfonic acids that disappeared with the aqueous phases.

**Scheme 3:**
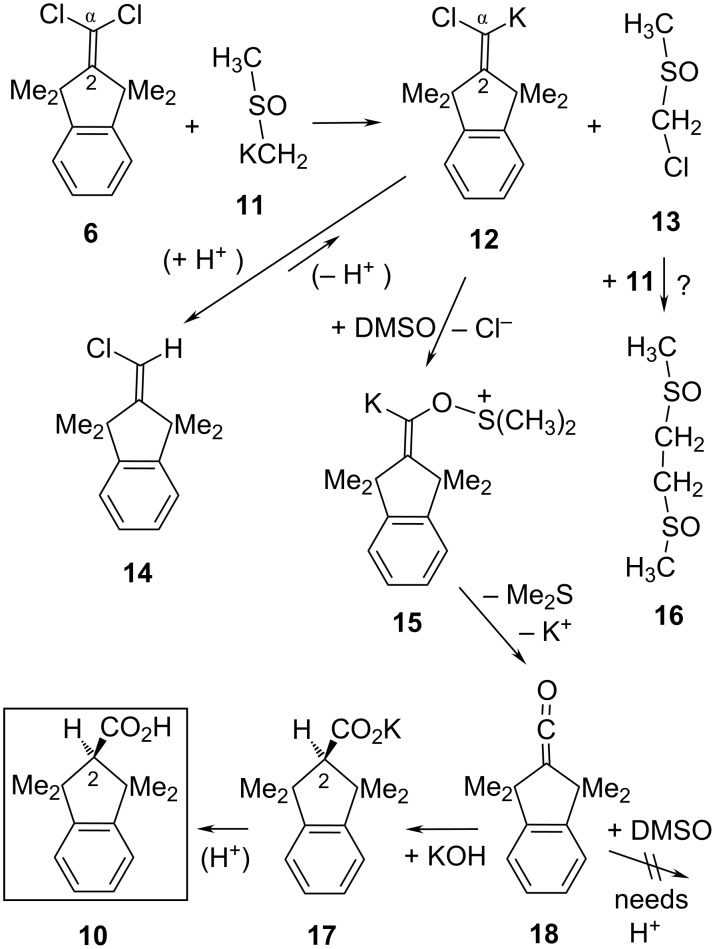
Proposed carbenoid pathway from **6** to acid **10** and dimethyl sulfide (Me_2_S) in DMSO as the solvent.

Without electron-withdrawing substituents in both **6** and the prospective ARE [12] intermediate **9**, several strongly caustic methods of hydrolysis with KOH failed to consume **6** in diethylene glycol [18] (11 hours at 135 °C), in triglyme (six hours, 150 °C), in HMPA [tris(dimethylamino)phosphinoxide, five hours at 150 °C], and in acetonitrile (23 hours, 70 °C). Reisolation of pure **6** from hot formic acid (44 hours, 90 °C) or from dimethyl sulfoxide solution (DMSO, five hours at 156 °C without a base) showed that C–Cl bond heterolysis (vinylic S_N_1 reaction) did not occur in these polar solvents. In DMSO with potassium *tert*-butoxide (KO*t-*Bu, four equiv, >22 hours at 140 °C) or better with solid KOH (at least 29 equiv, ≥ six hours at 100 °C or 60 min at 154 °C), however, **6** was slowly transformed into **10**. This may be reminiscent of a 10^9^-fold increased kinetic basicity [19–20] of KOCH_3_ in DMSO as a solvent. Due to the strongly enhanced thermodynamic basicity of solid KOH in DMSO [21–23], this system will provide and maintain a small concentration of the potassium salt **11** of DMSO (“dimsyl-K”, Scheme 2). On the other hand, a more special involvement of DMSO as an oxidant in the present “hydrolysis” of **6** became evident when pure dimethyl sulfide (Me_2_S, boiling point 37 °C) distilled from the reaction vessel into a cold trap during such a preparation of **10**. In situ ^1^H NMR spectra revealed the obligatory formation of ca. one equivalent of Me_2_S (δ_H_ = 2.06 ppm). Small portions of Me_2_S stemmed from the slow destruction of DMSO by **11**, as confirmed in a faster run with **11** alone in DMSO during four hours at 150 °C, and presumably also from generation of the side-product potassium formate as formulated further below. Clearly, Me_2_S could not have been formed from **6** in a simple hydrolytic ARE [12] process with an intermediate such as **9** as a precursor of **10**. Instead, we propose a carbenoid pathway in Scheme 3 and justify the specified steps in the sequel.

In a Cl/K interchange reaction that generates the Cl,K-carbenoid **12**, the transfer of a chlorine particle from **6** to KOH cannot be excluded at the outset in view of the early protocols [24–27] which described the application of dry NaOEt or KO*t-*Bu at ca. 190 °C for converting aryl_2_C=CCl_2_ into aryl_2_CH–CO_2_H along with aryl–C≡C–aryl (the latter in a Fritsch–Buttenberg–Wiechell (FBW) rearrangement [13]). However, dimsyl-K (**11**) in DMSO without KOH consumed **6** immediately already at rt, albeit without formation of the acid **10**. This suggests KOH to be essential for creating product **10**; it also suggests that KOH might be a poor competitor of **11** in a nucleophilic attack on **6**, perhaps due partially to the low solubility (ca. 0.001 M [21]) of KOH in DMSO. Granting preference to the chlorine transfer from **6** to **11**, the byproduct **13** of **12** would hardly be traceable: even if **13** reacted with **11**, for example, the resultant water-soluble [28] bis(sulfoxide) **16** might get lost in the usual procedure of aqueous work-up. As an important confirmation of the Cl,K-carbenoid **12**, however, its conjugated CH-acid **14** [29] was observed during the early period of a running transformation and vanished slowly with regeneration of **12**. This interpretation was corroborated through independent generations of **12** from **14**: Using suspensions of KO*t-*Bu as the base in warm THF or cyclohexane for deprotonating **14**, the generated **12** was found [30] to undergo expansion (FBW) of its five-membered ring rather than the intended formation of the acid **10**. As two demonstrations that FBW ring expansion is not an inevitable fate of bona-fide **12**, we deprotonated **14** also with the freely soluble base KN(SiMe_3_)_2_ (in place of the hardly soluble KO*t-*Bu) in *t-*BuOMe or in THF as the solvents and observed [30] in situ merely the nonexpanded enamine (the expected S_N_V product). Alternatively, we generated **12** from the dichloroalkene **6** with a THF solution of benzyl potassium, finding only the carbenoid chain (S_N_V) product and its descendants but again no ring expansion [30]. Thus, **12** behaves like a normal unsaturated carbenoid whose S_N_V reaction requires a more abundant, soluble nucleophile than solid KO*t-*Bu (or, by analogy, solid KOH). These observations pointed to a possible S_N_V reaction of **12** with DMSO and/or dimsyl-K (**11**), as proposed in Scheme 3 and later on as follows.

In a rapid oxygen-transfer reaction that is known [31–32] for saturated carbenes or carbenoids, **12** will attack the solvent DMSO to generate the K,O-carbenoid **15** which decays in an E1cb-like expulsion of the observed Me_2_S. The resultant, still unknown di-*tert*-alkyl ketene **18** appears to have only limited options in this milieu: Taking *t-*Bu_2_C=C=O [2] as a model of the slightly less congested ketene **18**, a Pummerer-type acylation of DMSO by **18** should require the assistance of a carboxylic acid [33]; this is excluded in our strongly basic system. On the other hand, KOH consumed *t-*Bu_2_C=C=O at rt readily [34]; thus, the certainly faster addition of KOH to **18** can give the observed (in situ ^1^H NMR) potassium salt **17** of **10**. Of course, [2-D]**17** was detected and [2-D]**10** isolated [30] (with ca. 80% D, for example) from runs of **6** with KOH in [D_6_]DMSO due to the H/D interchange reaction affording KOD; a separate experiment established that [2-D]**17** was not formed in a subsequent step from unlabeled **17** with **11** in [D_6_]DMSO in the course of 11 hours at 120 °C. In the outlined mechanism, the formal “hydrolysis” of **6** to produce the acid **10** can be seen to consist of an initial reduction of **6** generating the carbenoid **12**, followed by the oxidation of **12** by DMSO that affords the precursors of **10**.

Although the isolated yield of acid **10** never exceeded 60%, our preparation is convenient because **10** was obtained as the only acid and in a suitably clean state, expecially when the acidified alkaline layers of aqueous work-up were extracted with pentane (in place of Et_2_O) which can more efficiently be cleaned from the last traces of DMSO through washing with water. The procedure can also be made colleague-friendly by an early deodorizing of the Me_2_S contamination through a short treatment of the aqueous solution of **17** with NaOCl (but not KMnO_4_), to be terminated soon through the addition of NaHSO_3_. Gratifyingly, all side-products did not contaminate the acid **10** because they assembled in the nonacidic fraction as described in the sequel.

**Scheme 4:**
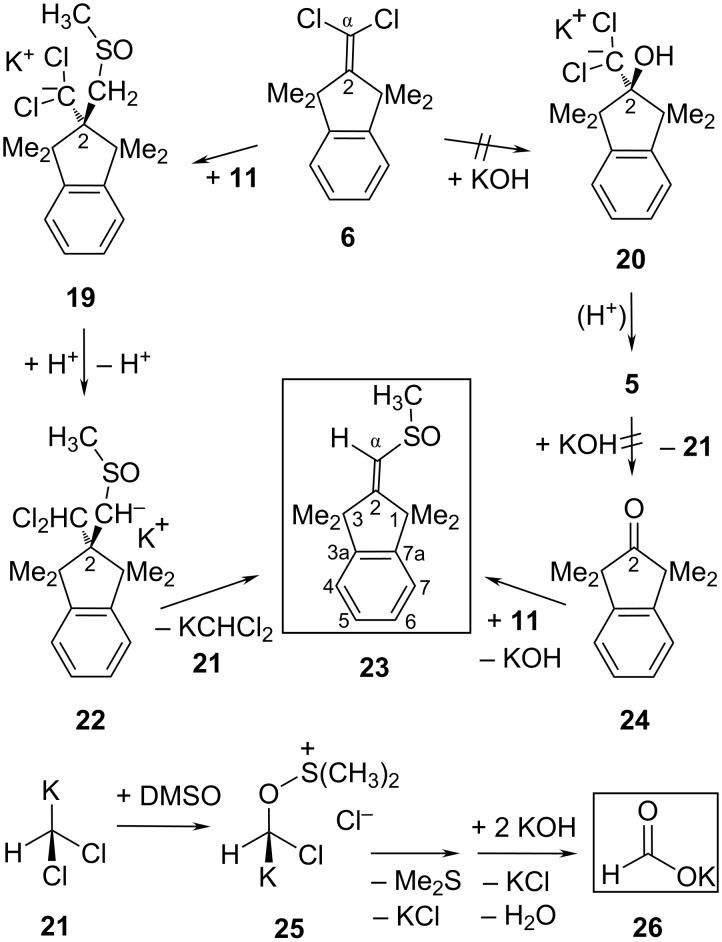
Proposed pathway to the main side-product **23** formed by nucleophilic addition of **11** to C-2 of **6**.

The structure of the main side-product **23** (Scheme 4) suggests that it might result from a base-catalyzed condensation of DMSO with 1,1,3,3-tetramethylindan-2-one (**24**) [35]; this assumption seemed confirmed through the isolation of **23** (57%) from **24** with KOH (28 equiv) in DMSO after 16 hours at 100 °C. However, the idea that **24** might have been generated from **6** via **20** (and the dichloro alcohol **5**) had to be dismissed: Although an authentic [3] sample of **5** was quickly consumed under the above conditions of hydrolyzing **6**, it furnished none of **24**, **23** or **10**. Nevertheless, **24** accumulated in the last period of transforming **6** into **10** (>15 hours at or above 100 °C); such a late appearance of **24** may be caused by a slowly emerging precursor (other than **5**). While **24** obviously did not hasten to form **23** with DMSO, an alternative route from **6** with **11** to **23** appears reasonable: the first intermediate **19** would isomerize to **22** and then dissociate into KCHCl_2_ (**21**) and product **23**. As a Cl,K-carbenoid, **21** will fall a victim to the above-mentioned type of oxygen-transfer reaction [31–32] with DMSO, via **25** for example, to furnish Me_2_S and potassium formate (**26**) which was actually detected (δ_H_ = 8.54 ppm) in situ.

**Scheme 5:**
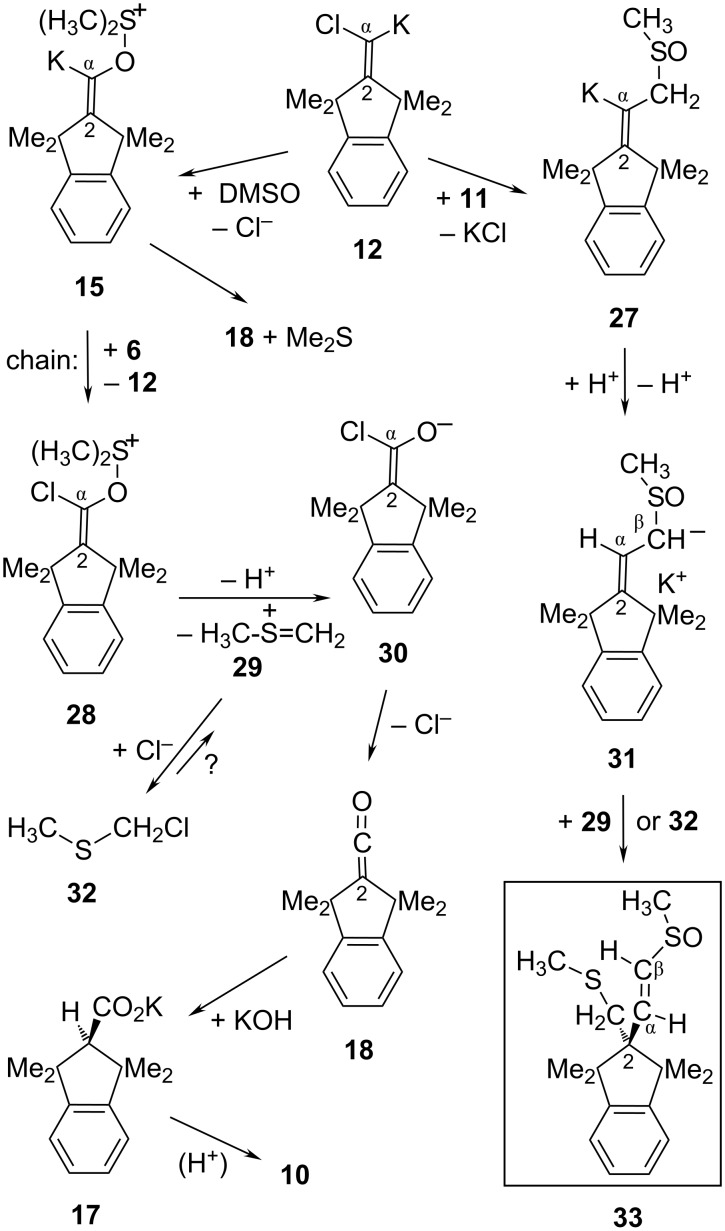
Possible course of the carbenoid *chain* reaction of **12** with DMSO and the α,α-dichloroalkene **6**.

The carbenoid chain [3] option has not yet been addressed in the above proposals, because the results outlined in Scheme 3 and Scheme 4 were not compatible with that variant. However, a further side-product **33** was encountered whose structure may reasonably be explained using the carbenoid chain mechanism in the following manner. As a competitor of the Me_2_S-producing decay of the K,O-carbenoid **15** to give **18** (Scheme 5), a chlorine particle transfer from **6** to **15** will close a carbenoid chain cycle and give rise to the chain carrier **12** together with the primary product **28**. In analogy with the base-induced O–S bond cleavage during the Pummerer acylation with *t-*Bu_2_C=C=O [36], a base-induced decay of **28** can be expected to generate the 2-thioniapropene **29** along with the α-chloroenolate **30** that may produce the ketene **18** (or the unknown acyl choride) and finally (as in Scheme 3) the precursor **17** of acid **10**. This alternative route to acid **10** does not create Me_2_S and may be a minor contribution, therefore. The expelled cation **29** may be consumed by the liberated chloride anion to give H_3_C–S–CH_2_Cl (**32**) or by dimsyl-K (**11**) or by formation of **33** along the following lines (Scheme 5).

In competition with the abundant DMSO to give **15**, dimsyl-K (**11**) may displace a chloride anion from the Cl,K-carbenoid **12** to generate the vinylpotassium derivative **27**. Proton transfer steps should isomerize **27** into the energetically more stable allyl anion derivative **31**. Either **29** or **32** may intercept **31** with formation of the side-product **33**, whose structure was recognized through its NMR data [30] as follows. The precursor **31** of **33** cannot have reacted at its C-β center, since this would have led to an isomer of **33** with only one proton-bearing olefinic ^13^C center (C-α), whereas two olefinic CH centers (C-α and C-β) were detected in **33** while its C-2 atom was found to be aliphatic rather than olefinic. Although **33** is chiral, its only C*H*_2_ signal exhibits merely weak line-broadening instead of the expected AB splitting pattern; this indicated the center of chirality (sulfur) to be rather distant from CH_2_. The NOESY correlation of this quasi-singlet C*H*_2_ signal with the SC*H*_3_ resonance established the presence of a CH_2_–S–CH_3_ instead of a CH_2_–S(O)–CH_3_ fragment. The =CH–S(O)–CH_3_ group and its proximity to the CH_2_ protons were recognized through the NOESY correlations of the olefinic β-proton. This completes the mechanistic considerations for the unusual “hydrolysis” reaction of the unactivated α,α-dichloroalkene **6** and its side-products.

### Toward one-sidedly overcrowded ketone and bromoalkene descendants

**Scheme 6:**
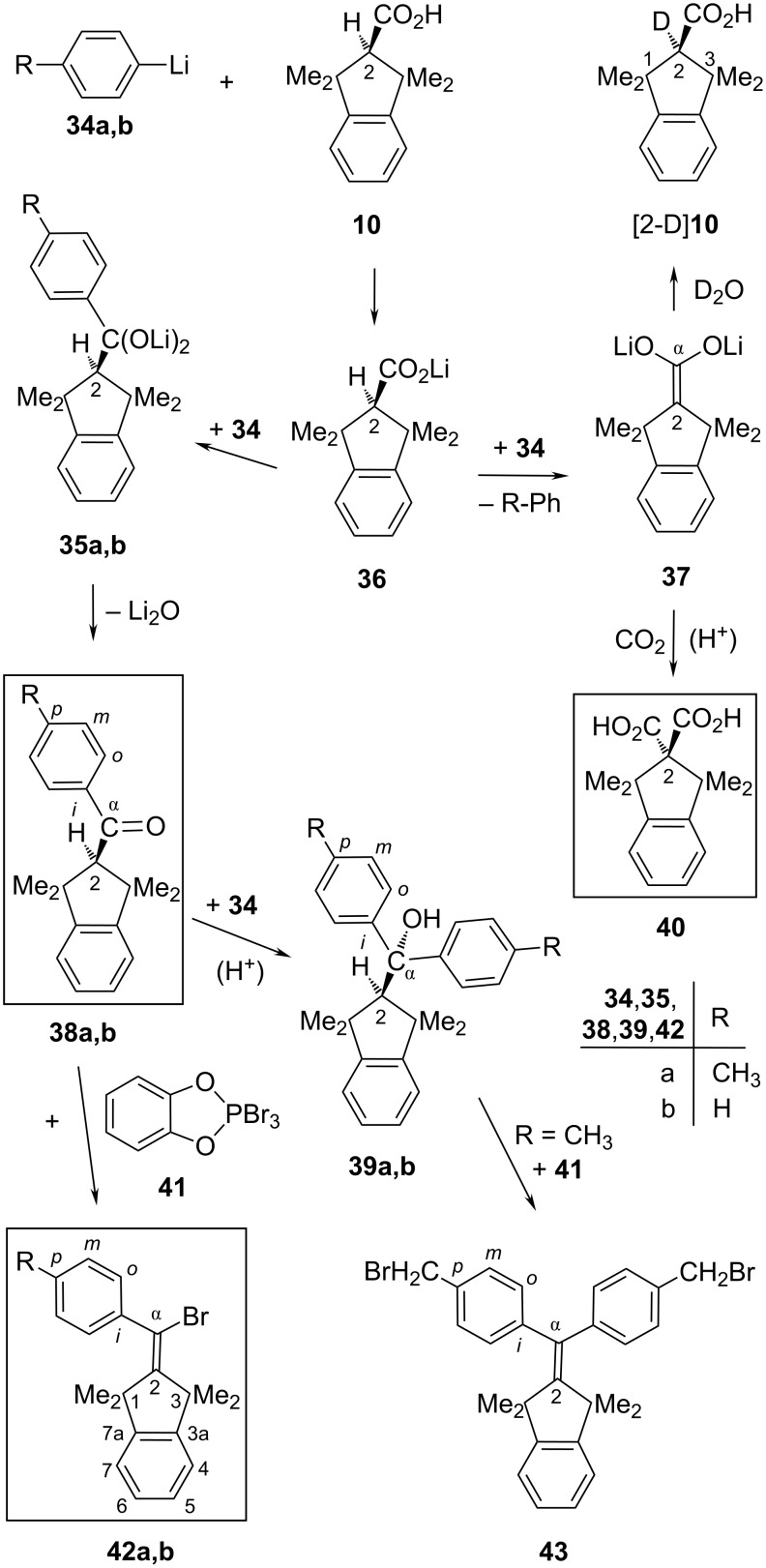
Synthesis of the one-sidedly overcrowded descendants **38**, **39**, and **42**.

The α,α-(di-*tert*-alkyl)methyl aryl ketones **38** (Scheme 6) would normally be considered to be accessible through Friedel–Crafts acylation of arenes by the acid chloride of **10**. This possibility was repudiated, however, because the similar acyl chloride *t-*Bu_2_CH–CO–Cl did not acylate benzene [37–38]. Instead, the technique [39] of acylating an aryllithium compound such as **34** by a lithium carboxylate such as **36** to form **35** proved effective in Et_2_O as the solvent, in spite of a dissuasive message [40] that was based on the failure with 2,6-dimethylbenzoic acid at ice temperature. Admittedly, our protocol requires prolonged stirring in Et_2_O at or above rt due to steric shielding in **36**, which enabled at least two yield-reducing side-reactions to consume the aryllithium reagents **34a**,**b** to some extent, as delineated below. Therefore, acid **10** was treated with an excess of **34a**,**b** which were generated from aryl bromides, using either 2.5 equivalents thereof together with *tert*-butyllithium (*t-*BuLi, five equiv) or four equivalents of both aryl bromide and *n-*BuLi. The preformed sodium carboxylate of **10** in place of **36** was apparently too insoluble in Et_2_O to react with **34**.

The primary products **35** tend to eliminate Li_2_O slowly with formation of ketones **38** in the presence of aryllithium reagents **34** which will rapidly add to **38**, creating the tertiary alcohols **39**. The unwanted side-products **39** might also arise during simple aqueous work-up where hydrolytic ketone formation from primary products such as **35** was often [40–41] able to compete with the fast protolysis of residual aryllithium reagents. To avoid this suspected mischief, we applied nonaqueous quenching of residual **34**, using either carboxylation by solid CO_2_ or the commended treatments with aniline [41] as the proton donor or with Me_3_SiCl [40] prior to the aqueous work-up. However, none of these three precautionary measures improved the **38**/**39** ratios (ca. 9:1 after ca. 20 hours at rt) significantly, which means that the side-products **39** arose essentially before the work-up procedure. Actually, the unwelcome portions of **39** increased in parallel with extending reaction periods and made especially the isolation of pure ketone **38b** somewhat tedious. (An alternative route [30] can provide **38b** without side-products.) The overcrowded nature of **39a** and **39b** became evident [30] through NMR line broadening effects as caused by impeded rotations about the C-α/C-*ipso* single bonds.

As a second side-reaction, deprotonation of 2-H in **36** by **34** competed with the retarded acylation of **34**, so that the dianion **37** of acid **10** was generated and contributed to the recovery of **10** on aqueous work-up. The intermediacy of **37** was recognized through quenching either with D_2_O to afford [2-D]**10** or with solid CO_2_ and subsequent isolation of the highly symmetric diacid **40** (only seven ^13^C NMR signals). The constitution of **40** followed from its ready decarboxylation in CDCl_3_ solution at rt to regenerate acid **10** within four days.

Conversion of the ketone **38a** into bromoalkene **42a** through brominative deoxygenation [42–43] with tribromide **41** was slow in hot chloroform but almost complete within 17 hours at 80 °C in 1,2-dichloroethane as the solvent. The purification of **42a** by simple recrystallization was easy, unless the employed ketone **38a** was contaminated with its side-product **39a** whose *p*-methyl groups were brominated by reagent **41** with formation of **43** [30]. The pure ketone **38b** and reagent **41** furnished the known [44–45] bromoalkene **42b** as the only product [30,46].

## Conclusion

The observed features of the “hydrolysis” reaction of the unactivated α,α-dichloroalkene **6** with solid KOH in DMSO are incompatible with the key intermediate **9** of the ARE [12] mechanism. Instead, a Cl,K-carbenoid **12** (indicated by the observation of its reversible protonation) is compatible and appropriate as the first intermediate in the route from **6** to acid **10**. This formal “hydrolysis” of **6** makes use of the impressively enhanced electrophilicity of “unsaturated” carbenoids [13] such as **12**, which property enables **12** to react with the moderately nucleophilic but abundant solvent DMSO that brings along the possibility of an oxygen transfer: The necessary cleavage of the O–S bond in the resultant K,O-carbenoid **15** may occur either directly (the Me_2_S producing pathway in Scheme 3) or upon formation of the carbenoid *chain* product **28** (Scheme 5). Obviously, DMSO is a unique choice for oxidizing **12** in both the chain and the nonchain mechanistic options. The nonoxidizing nucleophiles PhCH_2_K and KN(SiMe_3_)_2_ can convert **12** to the expected nonacidic S_N_V products, whereas **12** prefers to expand its five-membered ring if a suitable nucleophile is not available [30].

The easily isolated and purified acid **10** is able to transfer its bulky di-*tert*-alkylacetyl body onto a sufficiently reactive nucleophile. The first nucleophilic attack is already sterically impeded, as suggested by its ability to compete with the generation of dianion **37** of acid **10** (Scheme 6). Nevertheless, such one-sided shielding in the product ketone does not prevent the incorporation of a second nucleophile, affording a tertiary alcohol with a substantially impeded internal mobility. Final brominative deoxygenation of the ketones can yield bromoalkenes as the only products with moderately decreased speed.

## Experimental

**1,1,3,3-Tetramethylindan-2-carboxylic acid (10):** The following procedure should be carried out in an efficient hood and/or with a cold trap that can collect and retain malodorant volatile products such as Me_2_S. The dichloroalkene **6** (2.80 g, 11.0 mmol), dimethyl sulfoxide (45 mL), and a magnetic stirring bar were placed in a round-bottomed flask carrying either a wide-bore connection to a cooled trap or a T-shaped glass-tube with a bubbler and gas inlet. This suspension was stirred at 60 °C until **6** was completely dissolved. Freshly pulverized KOH (18.0 g, 321 mmol) was added and the brown mixture stirred at 100 °C under inert-gas cover for at least six hours, then cooled to rt, poured into distilled water (250 mL), and shaken with Et_2_O (3 × 50 mL). The malodorant aqueous layer containing the potassium salt **17** was treated batchwise with NaOCl solution in an amount that sufficed for showing a positive potassium iodide/starch test for 30 min, at which time this residual NaOCl was forthwith destroyed by solid NaHSO_3_ that had to be added until the KI/starch test became negative. (KMnO_4_ in place of NaOCl was found to decompose **17**.) The solution was thoroughly stirred with charcoal, then filtered, cooled in ice, and acidified with concd. hydrochloric acid. The precipitated acid **10** was extracted with Et_2_O or (better) pentane (3 × 70 mL). These combined extracts were repeatedly washed with distilled water to remove traces of DMSO, dried over Na_2_SO_4_, and evaporated to leave the almost pure acid **10** as a white powder (1.31 g, 54%) with mp 188–190 °C (ref [15]: 189–190.5 °C); ^1^H NMR (CDCl_3_, 400 MHz) δ 1.41 (s, 6H, 1-/3-CH_3_ syn to CO_2_H), 1.52 (s, 6H, 1-/3-CH_3_ anti to CO_2_H), 2.91 (s, 1H, 2-H), 7.16 (AA´ part of an AA´BB´ system, 2H, 4-/7-H), 7.24 (BB´ part, 2H, 5-/6-H) ppm, assigned through the NOESY correlations 2-H ↔ anti-CH_3_ ↔ syn-CH_3_ ↔ 4-/7-H ↔ anti-CH_3_; ^1^H NMR ([D_6_]acetone, 400 MHz) δ 1.35, 1.48, 2.85, 7.12, 7.18 ppm; ^13^C NMR (CDCl_3_, 100.6 MHz) δ 27.4 (1-/3-CH_3_ syn to CO_2_H), 30.2 (1-/3-CH_3_ anti to CO_2_H), 45.6 (C-1/-3), 64.8 (C-2), 122.3 (C-4/-7), 127.3 (C-5/-6), 149.4 (C-3a/7a), 179.1 (CO_2_H) ppm, assigned through HSQC; ^13^C NMR ([D_6_]acetone, 100.6 MHz) δ 27.7, 30.5, 45.9, 65.2, 123.1, 128.0, 150.5, 173.4 ppm.

**1,1,3,3-Tetramethyl-2-(methylsulfinylmethylidene)indane (23).** The combined Et_2_O extracts containing the nonacidic side-products, as obtained in the above preparation of **10** and separated from the alkaline aqueous layer, were washed with distilled water until neutral, dried over Na_2_SO_4_, and evaporated. The remaining brown solid (<977 mg) contained mainly the sulfoxides **23** and **33** (3:1) together with a little of 1,1,3,3-tetramethylindan-2-one (**24**). The pure sample of **23** (284 mg, 10%) was isolated through extraction into hot, low-boiling petroleum ether (60 mL), filtration, and concentration. Recrystallization afforded almost colorless needles with mp 134–135 °C; ^1^H NMR (CDCl_3_, 400 MHz) δ 1.41 and 1.45 (2 s, 2 × 3H, 2 × 3-CH_3_), 1.51 and 1.73 (2 s, 2 × 3H, 2 × 1-CH_3_), 2.67 (s, 3H, OS-CH_3_), 6.25 (s, 1H, α-H), 7.17 (AA´ part of an AA´BB´ system, 2H, 4-/7-H), 7.27 (BB´ part, 2H, 5-/6-H) ppm, assigned through comparison with the phenylsulfinylmethylidene analogue [47]; ^1^H NMR (DMSO, 200 MHz) δ 1.38 (2 × 3-CH_3_), 1.46 and 1.63 (2 × 1-CH_3_), 6.47 (α-H), 7.24 (quasi-s, 4-/5-/6-/7-H); ^13^C NMR (CDCl_3_, 100.6 MHz) δ 30.12 and 31.94 (2 × 3-CH_3_), 31.54 and 32.95 (2 × 1-CH_3_), 40.73 (OS–CH_3_), 48.54 (C-3), 48.76 (C-1), 122.41 and 122.51 (C-4/-7), 127.70 and 127.82 (C-5/-6), 128.78 (C-α), 147.04 (C-3a), 148.23 (C-7a), 174.13 (C-2) ppm, assigned as above in accord with the deuterium-induced shifts ^2^*Δ* = –0.068 for C-2, ^3^*Δ* = −0.021 for C-3, and ^3^*Δ* = −0.045 ppm for C-1 as caused by the =C^α^D–S(O)–CD_3_ group incorporated during a run in [D_6_]DMSO; IR (KBr) ν: 2971, 2959, 2920, 2860, 1627 (w), 1484, 1363, 1032, 1022, 968, 748, 677, 505 cm^−1^; anal. calcd for C_15_H_20_OS (248.39): C, 72.53; H, 8.12; S, 12.91; found: C, 72.75; H, 8.25; S, 12.94.

**2-(*p*-Methylbenzoyl)-1,1,3,3-tetramethylindane (38a):** A round-bottomed Schlenk flask (50 mL) was charged with 4-bromotoluene (0.563 mL, 4.58 mmol), anhydrous Et_2_O (10 mL), and a magnetic stirring bar. The contents were stirred and cooled at −78 °C under argon gas cover during the dropwise addition of *t-*BuLi (9.16 mmol) in pentane (6.10 mL), then stirred without cooling for 30 min. After the dropwise addition of acid **10** (400 mg, 1.83 mmol) in anhydrous Et_2_O (10 mL) to this solution of *p*-methylphenyllithium (**34a**) and further stirring at rt for 18 hours, the mixture was poured onto solid CO_2_, warmed up, and diluted with aqueous NaOH (1 M, 20 mL). The aqueous layer was shaken with Et_2_O (3 × 20 mL) and the combined four Et_2_O layers were washed with distilled water until neutral, dried over Na_2_SO_4_, and concentrated to leave the crude nonacidic material (455 mg) consisting mainly of **38a**, **39a**, and toluene (9:1:9). Repeated crystallizations from pentane afforded white needles of **38a** (isolated yield up to 35%); mp 95.5–96.5 °C (methanol); ^1^H NMR (CDCl_3_, 400 MHz) δ 1.34 and 1.39 (2 s, 2 × 6H, 2 × 1-/3-CH_3_), 2.42 (s, 3H, *p*-CH_3_), 4.10 (s, 1H, 2-H), 7.17 and 7.24 (AA´BB´ system, 2 × 2H, 4-/5-/6-/7-H), 7.28 (broadened d, ^3^*J* = 8.3 Hz, 2H, 2 × *m*-H), 7.88 (dm, ^3^*J* = 8.3 Hz, 2H, 2 × *o*-H) ppm; ^1^H NMR (CCl_4_, 80 MHz) δ 1.29, 1.35, 2.39, 3.99, 7.06 (s, 4H), 7.18 (d), 7.80 (d) ppm; ^13^C NMR (CDCl_3_, 100.6 MHz) δ 21.56 (*p*-CH_3_), 28.02 and 30.98 (2 × 1-/3-CH_3_), 47.17 (C-1/-3), 64.32 (C-2), 122.34 (C-4/-7), 127.12 (C-5/-6), 128.28 and 129.34 (2 × C-*m* and 2 × C-*o*), 138.36 (C-*ipso*), 143.50 (C-*p*), 149.99 (C-3a/7a), 201.25 (C=O) ppm; IR (KBr) ν: 2967, 2925, 2862, 1664 (s), 1606, 1480, 1368, 1228, 1209, 1186, 869, 759 cm^−1^; anal. calcd for C_21_H_24_O (292.42): C, 86.26; H, 8.27; found: C, 86.50; H, 8.41.

The above aqueous NaOH layer was acidified with concd. hydrochloric acid and was shaken with Et_2_O (3 × 10 mL). These combined Et_2_O extracts were washed with distilled water until neutral, dried over Na_2_SO_4_, and evaporated to leave a white powder containing acid **10**, *p*-methylbenzoic acid, *p*-cresol, and diacid **40** (ca. 4:2:2:1).

**1,1,3,3-Tetramethylindan-2,2-dicarboxylic acid (40):** The mixture of acids obtained above (see **38a**) was leached with pentane, which left the insoluble diacid **40** behind (65 mg, 14% yield). One recrystallization from hot toluene afforded clean **40** (30 mg) but led to the decarboxylation of a portion that remained dissolved. The transparent needles of pure **40** had a mp of 195–197.5 °C (dec.), decomposed slowly on standing at rt in CDCl_3_ solution, and were weakly soluble in CCl_4_ only as long as they were a part of the original mixture with the other carboxylic acids. ^1^H NMR ([D_6_]acetone or CDCl_3_, 400 MHz) δ 1.58 (s, 12H, 2 × 1-/3-CH_3_), 7.21 (quasi-s, 4H, 4-/5-/6-/7-H) ppm; ^13^C NMR ([D_6_]acetone, 100.6 MHz) δ 28.83 (2 × 1-/3-CH_3_), 48.98 (C-1/-3), 122.28 (C-4/-7), 125.79 (C-2), 127.59 (C-5/-6), 150.30 (C-3a/7a), 171.73 (2 × CO_2_H) ppm, assigned through comparison with the acid **10**; IR (KBr) ν: 3400–2500 (very broad H–O), 1713 (s), 1291, 759 cm^−1^; anal. calcd for C_15_H_18_O_4_ (262.3): C, 68.69; H, 6.92; found: C, 69.23; H, 6.81.

**40** was not formed from acid **10** with lithium *N*,*N*-diisopropylamide in THF as the solvent at rt: final quenching with solid CO_2_ afforded only starting material **10**.

**2-(α-Bromo-*p*-methylbenzylidene)-1,1,3,3-tetramethylindane (42a):** A dry NMR tube (5 mm) was charged under argon cover gas with 2-bromo-1,3,2-benzodioxaphosphole [48] (0.12 mL, 0.95 mmol) and anhydrous, EtOH-free 1,2-dichloroethane (0.5 mL). The solution was cooled in ice, treated with elemental bromine (0.040 mL, 0.78 mmol), and kept at rt for 15 min. This yellow solution of 2,2,2-tribromo-2,2-dihydro-1,3,2-benzodioxaphosphole (**41**) [49] was recooled in ice for the addition of the pure, solid ketone **38a** (200 mg, 0.68 mmol), which caused an immediate color change to brown. The tightly stoppered tube was heated at 80 °C for at least 17 hours, then diluted with pentane (5 mL), and poured onto ice-cooled aqueous Na_2_CO_3_ solution (2 M, 5 mL), which was stirred for 15 min. The aqueous Na_2_CO_3_ layer was shaken with pentane (2 × 5 mL) and discarded. The combined pentane extracts were shaken with aqueous NaOH (2 M, 3 × 5 mL), washed with distilled water (10 mL), dried over CaCl_2_, and evaporated to furnish the solid nonacidic fraction (256 mg) containing a little residual ketone **38a**. Recrystallization from ethanol (12 mL) yielded pure needles of **42a** (173 mg, 71%), mp 119.5–121 °C; ^1^H NMR (CDCl_3_, 400 MHz) δ 1.19 (s, 6H, 2 × 1-CH_3_), 1.78 (s, 6H, 2 × 3-CH_3_), 2.38 (s, 3H, *p*-CH_3_), 7.03 (dm, ^3^*J* = 7.5 Hz, 1H, 7-H), 7.16 and 7.22 (quasi-AB system, ^3^*J* = 8 Hz, 2 × 2H, 2 × *o*-H and 2 × *m*-H of tolyl), 7.18–7.27 (m, 3H, 4-/5-/6-H) ppm, assigned through comparison with **42b** [45]; ^1^H NMR (CCl_4_, 80 MHz) δ 1.17, 1.76, 2.38, 7.14 ppm; ^13^C NMR (CDCl_3_, 100.6 MHz) δ 21.30 (*p*-CH_3_), 28.05 (2 × 3-CH_3_), 31.19 (2 ×1-CH_3_), 50.55 (C-1/-3), 117.95 (C-α), 122.10 (C-4), 122.59 (C-7), 127.16 (C-5), 127.24 (C-6), 128.53 (2 × C-*m*), 129.61 (2 × C-*o*), 137.66 (C-*p*), 139.93 (C-*ipso*), 149.00 (C-3a), 149.86 (C-7a), 157.11 (C-2) ppm, assigned as above; IR (KBr) ν: 2987, 2959, 2924, 2865, 1505, 1487, 1456, 1363, 799, 757 cm^−1^; anal. calcd for C_21_H_23_Br (355.32): C, 70.99; H, 6.52; Br, 22.49; found: C, 70.88, H, 6.66; Br, 23.17.

## Supporting Information

File 1 Alternative synthesis of ketone **38b**; preparation of [2-D]**10**, **33**, **39a**, **39b**, **42b**, and **43**; FBW ring expansion of carbenoid **12**; S_N_V reactions of **12** with PhCH_2_K and with KN(SiMe_3_)_2_.

## References

[1] Knorr R, Menke T, Ferchland K (2013). Organometallics.

[2] Knorr R, Hennig K-O, Schubert B, Böhrer P (2010). Eur J Org Chem.

[3] Knorr R, Pires C, Behringer C, Menke T, Freudenreich J, Rossmann E C, Böhrer P (2006). J Am Chem Soc.

[4] Pilati T, Simonetta M (1984). Acta Crystallogr, Sect C.

[5] Knorr R, von Roman T, Nöth H, Böck S (1992). J Chem Soc, Perkin Trans 2.

[6] Polborn K, Knorr R, Böhrer P (1992). Acta Crystallogr, Sect C.

[7] Knorr R, Hoang T P, Nöth H, Linti G (1992). Organometallics.

[8] Knorr R, Freudenreich J, Polborn K, Nöth H, Linti G (1994). Tetrahedron.

[9] Knorr R, Menke T, Ferchland K, Mehlstäubl J, Stephenson D S (2008). J Am Chem Soc.

[10] Knorr R, Menke T, Behringer C, Ferchland K, Mehlstäubl J, Lattke E (2013). Organometallics.

[11] Knorr R, Ruhdorfer J, Mehlstäubl J, Böhrer P, Stephenson D S (1993). Chem Ber.

[12] Rappoport Z (1992). Acc Chem Res.

[13] Knorr R (2004). Chem Rev.

[14] Knorr R, Pires C, Freudenreich J (2007). J Org Chem.

[15] Knorr R, Freudenreich J, von Roman T, Mehlstäubl J, Böhrer P (1993). Tetrahedron.

[16] Bott K, Hellmann H (1966). Angew Chem.

[17] Bott K (1967). Chem Ber.

[18] Grummit O, Buck A, Egan R (1955). Org Syn Coll Vol III.

[19] Cram D J, Rickborn B, Knox G R (1960). J Am Chem Soc.

[20] Cram D J, Rickborn B, Kingsbury C A, Haberfield P (1961). J Am Chem Soc.

[21] Dietrich B, Lehn J M (1973). Tetrahedron Lett.

[22] Finkentey C, Langhals E, Langhals H (1983). Chem Ber.

[23] Langhals E, Langhals H (1990). Tetrahedron Lett.

[24] Fritsch P, Feldmann F (1899). Justus Liebigs Ann Chem.

[25] Staudinger H, Rathsam G (1922). Helv Chim Acta.

[26] Harris E E, Frankforter G B (1926). J Am Chem Soc.

[27] Kaufman R J, Sidhu R S (1982). J Org Chem.

[28] Hull C M, Bargar T W (1975). J Org Chem.

[R29] 29Compound **2a** in ref [3].

[R30] 30See the Supporting Information File 1.

[31] Oda R, Mieno M, Hayashi Y (1967). Tetrahedron Lett.

[32] Tezuka Y, Miya M, Hashimoto A, Imai K (1987). J Chem Soc, Chem Commun.

[33] Knorr R (2011). Eur J Org Chem.

[R34] 34See p 6339 of ref [33].

[35] Knorr R, Mehlstäubl J, Böhrer P (1989). Chem Ber.

[R36] 36See the Schemes 2, 3, or 5 of ref [33].

[R37] 37See Scheme 4 of ref [33].

[38] Schaumann E, Walter W (1974). Chem Ber.

[39] Jorgensen M J (1970). Org React.

[40] Rubottom G M, Kim C (1983). J Org Chem.

[41] Nicodem D E, Marchiori M L P F C (1981). J Org Chem.

[42] von Roman U, Ruhdorfer J, Knorr R (1993). Synthesis.

[43] Behringer C, Knorr R (1997). J Prakt Chem.

[44] Knorr R, Lattke E, Räpple E (1980). Liebigs Ann Chem.

[45] Knorr R, von Roman T, Freudenreich J, Hoang T P, Mehlstäubl J, Böhrer P, Stephenson D S, Huber H, Schubert B (1993). Magn Reson Chem.

[R46] 46Compound **11j** in ref [42].

[R47] 47Compound **18** in ref [45].

[R48] 48Compound **5a** in ref [42].

[R49] 49Compound **6a** in ref [42].

