# Supplementary material for: Carbenoid-mediated nucleophilic “hydrolysis” of 2-(dichloromethylidene)-1,1,3,3-tetramethylindane with DMSO participation, affording access to one-sidedly overcrowded ketone and bromoalkene descendants§
Source: Beilstein J Org Chem. 2014 Jan 31;10:307–15. doi: 10.3762/bjoc.10.28 (PMC3944619; doi:10.3762/bjoc.10.28)
Supplement: File 1 — Alternative synthesis of ketone 38b; preparation of [2-D]10, 33, 39a, 39b, 42b, and 43; FBW ring expansion of carbenoid 12; SNV reactions of 12 with PhCH2K and with KN(SiMe3)2. [file Beilstein_J_Org_Chem-10-307-s001.pdf]

Supporting Information

for

Carbenoid-mediated nucleophilic “hydrolysis” of 2-(dichloromethylidene)-1,1,3,3-tetramethylindane with DMSO participation, affording access to one-sidedly overcrowded ketone and bromoalkene descendants

Rudolf Knorr*, Thomas Menke, Johannes Freudenreich and Claudio Pires

Address: Department Chemie, Ludwig-Maximilians-Universität München, Butenandtstrasse 5–13 (Haus F), 81377 München, Germany

Email: Rudolf Knorr* - rhk@cup.uni-muenchen.de
*Corresponding author

Alternative synthesis of ketone 38b; preparation of [2-D]10, 33, 39a, 39b, 42b, and 43; FBW ring expansion of carbenoid 12; S_NV reactions of 12 with $PhCH_2K$ and with $KN(SiMe_3)_2$

Contents:

1. An alternative route to ketone **38b** page S2

2. Products [2-D]**10**, **33**, **39a**, **39b**, **42b**, and **43** page S6

3. Alternative generations and the behavior of the Cl,K-carbenoid **12** page S10

3.1. FBW ring expansion of **12** page S10

3.2. The S_NV reaction of benzyl potassium (**S11**) with carbenoid **12** page S11

3.3. No ring expansion during deprotonation and S_NV of monochloride **14** by the potassium amide KHMDS page S13

4. References used in the Supporting Information page S15

1. An alternative route to ketone 38b

Epoxidation of the known [S1] olefin **S1** with peracetic acid furnished the oxirane **S2** (Scheme S1) whose chiral nature became immediately evident through the ^{1}H NMR nonequivalence of all four methyl groups. We did not succeed in the following two methods of ring-opening nucleophilic bromination of **S2** with the intention to arrive at the bromoalkene **42b**. Treatment with Li_2NiBr_4 in THF at rt [S2] led to the quantitative recovery of **S2**, whereas ring-opening by acetyl bromide plus $Et_4N^+Br^-$ in $CHCl_3$ or by pyridinium bromide in 1,2-dichloroethane at 100 °C furnished 1,1,2,3-tetramethylindene (**S3**) and benzaldehyde; this provided further examples of the imminent rearrangement [S3] in the 1,1,3,3-tetramethylindane system. Fortunately, deprotonation at C-2′ of **S2** by *n*-BuLi occurred readily in THF as the solvent at rt (but very slowly in hexane with a first half-reaction time of >50 hours). The resultant lithium enolate **S4** was trapped with $ClSiMe_3$ to give the α-$OSiMe_3$ derivative **S5**. Alternatively, the same procedure of generating **S4** but trapping by protonation afforded the ketone **38b**.

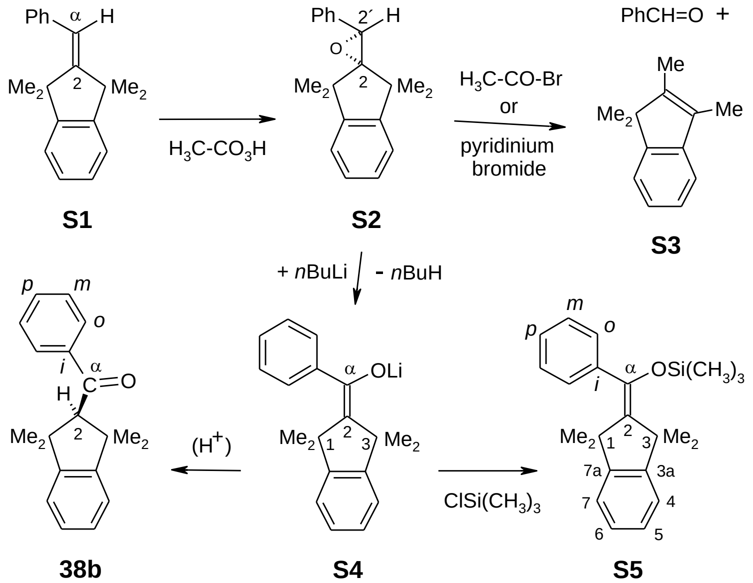

Scheme S1: An alternative synthesis of ketone **38b**.

1,1,3,3-Tetramethylspiro[2´-phenyloxirane-3´,2-indane] (S2): [S4] A suspension of the olefin **S1** (1.80 g, 6.86 mmol) [S1], suspended in glacial acetic acid (8 mL), was stirred at rt during the slow addition of peracetic acid (freshly prepared from H_2O_2 with acetanhydride). This batchwise addition was continued until the olefin **S1** had dissolved completely and a peroxide test (KI/starch) remained positive, which required several hours. The mixture was diluted with water (40 mL) and stirred for four hours to hydrolyze residual acetanhydride, then rinsed with more water (80 mL) and Et_2O (50 mL) into a separatory funnel. The aqueous layer was extracted with Et_2O (3×) and then discarded. The combined four Et_2O layers were washed with water (2×), aqueous NaOH (2 M, 2×) until the aqueous phase remained alkaline, again with water until neutral, and dried over Na_2SO_4. The filtered Et_2O solution was concentrated and dried in the presence of solid KOH in vacuo to afford the almost pure product **S2** (1.82 g, 95%). After a trap-to-trap distillation at 115–150 °C (bath temp.)/0.02 Torr, analytically pure **S2** was a colorless, viscous material that crystallized very slowly; mp 57–60 °C; ^{1}H NMR ($CDCl_3$, 80 MHz) δ 0.88, 1.13, 1.19, and 1.45 (4 s, 4 $\times$ 3H, 2 $\times$ 1-/3-CH_3), 4.07 (s, 1H, 2´-H), 6.90–7.40 (m, 9H, all aromatic H) ppm; ^{1}H NMR (CCl_4, 80 MHz) δ 0.86, 1.10, 1.15, 1.41, 3.95, 6.80–7.35 ppm; IR (film) ν: 3090, 3060, 3030, 2960 (s), 2925 (s), 2865, 1607 (w), 1590 (w), 1482, 1450, 1384, 950, 873, 746, 700 cm^{-1}; UV–vis (cyclohexane) λ_{max} (log ε) 271 (3.05), 264 (3.03, with vibrational progression by 980 cm^{-1}), 215 (4.29), 211 (4.22) nm; anal. calcd for $C_{20}H_{22}O$ (278.39): C, 86.29; H, 7.96; found: C, 86.64, H, 8.14.

2-[α-(Trimethylsilyloxy)benzylidene]-1,1,3,3-tetramethylindane (S5): [S5] The oxirane **S2** (460 mg, 1.65 mmol), anhydrous THF (10 mL), and a magnetic stirring bar were placed in a two-necked, round-bottomed flask (25 mL) fitted with a small pressure-equalizing dropping funnel carrying a gas bubbler. The solution was cooled

under nitrogen gas cover with stirring at −50 °C during the dropwise addition of *n*BuLi (4 mmol) in hexane (2.00 mL). After 60 min at −50 °C and further stirring at rt for at least 30 min, the yellow solution of the enolate **S4** (the in situ ^{1}H NMR showing a multiplet at δ_H = 6.65–7.13 and a quasi-singlet at 7.01 ppm) was cooled in ice and treated with $ClSiMe_3$ (0.50 mL). After one hour at rt, the mixture was diluted with Et_2O (30 mL) and poured into distilled water. The aqueous layer (pH = 10) was extracted with Et_2O until colorless, then discarded. The combined Et_2O layers were washed with distilled water until neutral, dried over Na_2SO_4, and evaporated to give an orange-colored solid (700 mg) consisting of **S5** mainly. Filtration and concentration of the hot solution of this solid in hexane furnished colorless crystals (400 mg, 69%) of pure **S5**; mp 96–97 °C (2 × from hexane); ^{1}H NMR ($CDCl_3$, 80 MHz) δ −0.07 (s, 9H, $OSiMe_3$), 1.11 (s, 6H, 2 × 1-CH_3), 1.60 (s, 6H, 2 × 3-CH_3), 6.95–7.27 (m, ca. 4H, 4-/5-/6-/7-H), 7.27 (quasi-s, 5H, α-phenyl) ppm, assigned as published [S6]; ^{13}C NMR as published [S6]; UV–vis (hexane) λ_{max} 270.4, 263.3 nm with vibrational progression by ca. 960 cm^{-1}; IR (KBr) ν: 2991, 2956, 2921, 2860, 1662, 1483, 1357, 1291, 1253, 1130, 1091, 1054, 1025, 910, 870, 864, 758, 705 cm^{-1}; anal. calcd for $C_{23}H_{30}OSi$ (350.58): C, 78.80; H, 8.62; found: C, 78.77; H, 8.68. **S5** is relatively stable against desilylating reagents.

2-Benzoyl-1,1,3,3-tetramethylindane (38b): *a) From the oxirane **S2***: [S5] The procedure described above for **S5** was repeated but the treatment with $ClSiMe_3$ was omitted: Colorless crystals; mp 82–84 °C (methanol); ^{1}H NMR ($CDCl_3$, 80 MHz) δ 1.29 and 1.33 (2 s, 2 × 6H, 2 × 1-/3-CH_3), 4.05 (s, 1H, 2-H), 7.14 (narrow m, 4H, 4-/5-/6-/7-H), 7.42 (m, 3H, *p*-H and 2 × *m*-H), 7.90 (m, 2H, 2 × *o*-H) ppm; IR (KBr) ν: 2960, 2920, 2859, 1673, 1663, 1448, 1370, 1218 (s), 760 cm^{-1}; anal. calcd for $C_{20}H_{22}O$ (278.39): C, 86.29; H, 7.96; found: C, 86.07; H, 7.94.

Without rigorous exclusion of air from the THF solution containing the enolate **S4** and *n*-BuLi, a side-product of uncertain constitution may appear and can be separated from the more soluble ketone **38b** through leaching with a hot alkane solvent: Colorless crystals; mp 132–135 °C (cyclohexane); ^{1}H NMR ($CDCl_3$, 80 MHz) δ 1.43 and 1.53 (2 s, 2 $\times$ 6H, 2 $\times$ 1-/3-CH_3), 6.90–7.41 (m, ca. 7H), 7.85 (m, 2H) ppm. This $CDCl_3$ solution decayed slowly at rt with formation of a singlet a δ = 1.31 ppm.

*b) From acid **10***: *n*-BuLi (7.33 mmol) in hexanes (3.46 mL) was added dropwise under argon gas cover to a stirred solution of bromobenzene (0.770 mL, 7.33 mmol) in anhydrous Et_2O (5.0 mL) cooled to −70 °C. After further stirring at rt for 60 min, this solution of phenyllithium (**34b**) and *n*-BuBr was added dropwise at rt to a stirred solution of acid **10** (400 mg, 1.83 mmol) in Et_2O (10 mL). Upon further stirring for 24 hours with exclusion of air and moisture, the yellow mixture containing a white precipitate was poured onto solid CO_2, warmed up, and dissolved in aqueous NaOH (1 M, 20 mL). The aqueous layer was shaken with Et_2O (3 × 20 mL) and the combined four Et_2O layers were washed with distilled water until neutral, dried over Na_2SO_4, and concentrated. This crude, non-acidic fraction (481 mg) contained the ketone **38b**, *n*-butylbenzene, and the alcohol **39b** in a 9:4:1 ratio. (The pure ketone **38b** was prepared by the alternative route described above.) The above aqueous NaOH layer was cooled in ice and acidified with conc. hydrochloric acid (white precipitate), then shaken with Et_2O (3 × 20 mL). These latter Et_2O extracts were combined and washed with distilled water until neutral, dried over Na_2SO_4, and evaporated to yield the acidic product fraction (222 mg) containing **10**, benzoic acid, and diacid **40** in roughly equal amounts.

2. Products [2-D]10, 33, 39a, 39b, 42b, and 43

[2-D]-1,1,3,3-Tetramethylindan-2-carboxylic acid ([2-D]10): This was isolated from a smaller run of the dichloroalkene **6** in [D_6]DMSO after 27 hours at 135 °C and exhibited the following deuterium-induced ^{1}H (400 Mz) and ^{13}C (100.6 MHz) NMR shifts $^n\Delta = \delta$([2-D]**10**) – δ(**10**), transmitted over n bonds in $CDCl_3$ as the solvent: 1-/3-C*H*$_3$ syn to CO_2H, $|^4\Delta|$ = ≤0.002; 1-/3-C*H*$_3$ anti, $^4\Delta = -0.0043$; 1-/3-*C*H$_3$ syn, $|^3\Delta|$ = <0.016; 1-/3-*C*H$_3$ anti, $^3\Delta = -0.052$; C-1/-3, $^2\Delta = -0.048$; C-2, $^1\Delta = -0.448$ ppm; $^1J_{C,D}$ = 19.4 Hz.

1,1,3,3-Tetramethyl-2-(2-methylsulfinylethen-1-yl)-2-(methylthiomethyl)indane (33): A small amount of this somewhat unstable side-product was isolated through crystallization (pentane) from the nonacidic fraction obtained in a larger (15 mmol) run. The resultant platelets (mp 134–135 °C, fortuitously the same mp as **23**) were identified beyond doubt by NMR techniques but could not be recrystallized. ^{1}H NMR ($CDCl_3$, 400 MHz) δ 1.21 (s, 3H, 1- or 3-CH_3 anti$_1$ relative to CH_2), 1.28 (s, 3H, 3- or 1-CH_3 anti$_2$), 1.39 (s, 3H, 1- or 3-CH_3 syn$_1$), 1.41 (s, 3H, 3- or 1-CH_3 syn$_2$), 2.19 (s, 3H, CH_3S), 2.59 (s, 3H, CH_3SO), 2.96 (quasi-s with linewidth 1.6 Hz, 2H, S–CH_2), 6.03 (broadened d, ^3J = 15.8 Hz, 1H, α-H), 6.74 (d, ^3J = 15.8 Hz, 1H, β-H), 7.10 (AB part of an ABMM´ system, 2H, 4-/7-H), 7.22 (MM´ part, 2H, 5-/6-H) ppm, assigned through the NOESY correlations H_3C–S $\leftrightarrow$ S–CH_2 (the only two-proton signal, correlates also with all four 1-/3-CH_3) $\leftrightarrow$ β-H $\leftrightarrow$ H_3C–SO, and β-H $\leftrightarrow$ α-H $\leftrightarrow$ both anti-1-/3-CH_3 $\leftrightarrow$ 4-/7-H; ^{13}C NMR ($CDCl_3$, 100.6 MHz) δ 18.0 (qt, ^1J = 138.0 Hz, ^3J = 2.4 Hz, H_3C–S), 26.28 (qq, 3- or 1-CH_3 anti$_2$), 26.31 (qq, 1- or 3-CH_3 anti$_1$), 29.19 (qq, 3- or 1-CH_3 syn$_2$), 29.39 (qq, 1- or 3-CH_3 syn$_1$), all of these four 1-/3-CH_3 having ^1J = 126.3 Hz and ^3J = 4.2 Hz, 36.37 (tqi, ^1J = 138.0 Hz, ^3J = 4.9 Hz, S–CH_2), 41.10 (qt, ^1J = 138.2 Hz, apparent t $J \approx 1.5$ Hz, H_3C–SO), 49.35 and 49.46 (unresolved, C-

1/-3), 59.06 (m, C-2), 122.74 and 122.81 (2 dm, ^1J = 156 Hz, C-4/-7), 127.50 and 127.51 (2 ddm, ^1J = 159.5 Hz, ^3J = 7.4 Hz, C-5/-6), 132.32 (dqi, ^1J = 173.5 Hz, apparent qi J = 4.2 Hz, C-β), 142.64 (dt, ^1J = 155.5 Hz, ^3J = 5.5 Hz, C-α), 148.72 (m, C-3a/-7a) ppm, assigned through HSQC spectra and the $J_{C,H}$ coupling constants.

p**,*****p*****´-Dimethyl-α-(1,1,3,3-tetramethylindan-2-yl)benzhydrol (39a):** As described for ketone **38a**, acid **10** (1.83 mmol) and *p*-methylphenyllithium (**34a**, from *n*BuLi, 7.33 mmol) but in refluxing Et_2O (15 mL, 48 hours at 40 °C) provided a 1:1 mixture of **38a** and **39a** which deposited **39a** from pentane as the solvent. The thin needles of pure **39a** had a mp of 189.5–191 °C; ^{1}H NMR ($CDCl_3$, 400 MHz, 25 °C) δ 1.01 and 1.33 (2 s, 2 × 6H, 2 × 1-/3-CH_3), 2.28 (s, 6H, 2 × *p*-CH_3), 2.84 (s, 1H, variable OH), 3.42 (s, 1H, 2-H), 7.05 and 7.17 (AA´BB´ system, 2 × 2H, 4-/5-/6-/7-H), 7.08 (dm, ^3J = 8.3 Hz, 4H, 2 × 2 *m*-H), 7.65 (d, ^3J = 8.3 Hz, 4H, 2 × 2 *o*-H) ppm; ^{1}H NMR ($CDCl_3$, 400 MHz, −56 °C) δ 1.01 and 1.37 (broadened), 2.30, 2.97, 3.44, 7.12 and 7.26 (AA´BB´ system), ca. 7.14 (d, 2 × 2 *m*-H), 7.65 and 7.72 (2 d, ^3J = 8 Hz, 2 × 2 *o*-H) ppm; ^{13}C NMR ($CDCl_3$, 100.6 MHz, 25 °C) δ 20.84 (*p*-CH_3), 29.46 and 33.54 (2 × 1-/3-CH_3), 47.23 (C-1/-3), 65.30 (C-2), 80.36 (HO–C), 122.00 (C-4/-7), 125.89 (broad, 2 × 2C-*o*), 126.80 (C-5/-6), 128.34 (2 × 2C-*m*), 135.77 (2C-*p*), 145.03 (2C-*ipso*), 150.52 (C-3a/-7a) ppm, assigned through comparison with benzyl alcohol; ^{13}C NMR ($CDCl_3$, 100.6 MHz, –56 °C) δ 20.96 (*p*-CH_3), 29.48 and 33.14 (broadened, 2 × 1-/3-CH_3), 46.91, 64.46, 79.84, 122.03, 123.30 and 127.78 (2 × 2C-*o*), 126.77 (C-5/-6), 127.94 and 128.59 (2 × 2C-*m*), 135.81, 144.32, 150.10 ppm; IR (KBr) ν: 3647 (sharp H–O), 2960, 1506, 1484, 1366, 796, 752 (s), 580 cm^{-1}; anal. calcd for $C_{28}H_{32}O$ (384.56): C, 87.45; H, 8.39; found: C, 87.69; H, 8.26.

As shown above for **39a** at −56 °C in $CDCl_3$ solution, the diastereotopic *o*-H, C-*o*, and C-*m* nuclei of the α,α-diaryl groups became pairwise chemically nonequivalent, while

the signals of the enantiotopic nuclei (p-CH_3, C-p, C-*ipso*, and p-CH_3) did not split. This established a restricted mobility at C-α with impeded rotation about the C-α/C-*ipso* single bonds and implies that the formation of **39a** and **39b** was retarded by a substantially increasing repulsive resistance.

α-(1,1,3,3-Tetramethylindan-2-yl)benzhydrol (39b): A run with acid **10** (1.83 mmol) and phenyllithium (**34b**, as in Section 1 for **38b** from *n*-BuLi, 7.33 mmol) in refluxing Et_2O (15 mL, six hours at 40 °C) provided a 2:1 mixture of **38b** and **39b**. A sample was crystallized from methanol and recrystallized from petroleum ether to give thin needles of pure **39b**, mp 205–206 °C; ^{1}H NMR ($CDCl_3$, 400 MHz) δ 1.01 and 1.34 (2 s, 2 × 6H, 2 × 1-/3-CH_3), 2.91 (s, 1H, variable OH), 3.48 (s, 1H, 2-H), 7.06 and 7.18 (AA´BB´ system, 2 × 2H, 4-/5-/6-/7-H), 7.17 (tt, ^3J = 7.7 Hz, 2H, 2 × p-H), 7.29 (t, ^3J = 7.9 Hz, 4H, 2 × 2 m-H), 7.81 (d, ^3J = 8.0 Hz, 4H, 2 × 2 o-H) ppm; ^{1}H NMR (CCl_4, 80 MHz) δ 0.98, 1.31, 2.75, 3.45, 7.12 (broad m, 10 H), 7.77 ppm; ^{13}C NMR ($CDCl_3$, 100.6 MHz) δ 29.46 and 33.52 (2 × 1-/3-CH_3), 47.26 (C-1/-3), 65.34 (C-2), 80.51 (HO–C), 122.01 (C-4/-7), 126.14 (very broad, 2 × 2C-*o*), 126.40 (2 × C-*p*), 126.88 (C-5/-6), 127.67 (broadened, 2 × 2C-*m*), 147.65 (2 × C-*ipso*), 150.38 (C-3a/-7a) ppm, assigned through comparison with benzyl alcohol; IR (KBr) ν: 3643 (sharp H–O), 3591 (w, H–O), 2982, 2958, 2867, 1486, 1447, 1366, 1065, 1029, 766, 711, 585 cm^{-1}; anal. calcd for $C_{26}H_{28}O$ (356.51): C, 87.60; H, 7.92; found: C, 87.92; H, 8.27.

2-(α-Bromobenzylidene)-1,1,3,3-tetramethylindane (42b): The brominative deoxygenation of ketone **38b** by reagent **41** (1.25 equiv) was conducted as described for **42a** but in EtOH-free CH_2Cl_2 in place of 1,2-dichloroethane as the solvent. The half-reaction time was ca. 48 hours at 47 °C with **42b** as the only product, while complete conversion had previously [S7] been attained within 75 hours at 60 °C in chloroform as the solvent. ^{1}H NMR ($CDCl_3$, 80 MHz) δ 1.17 (s, 6H, 2 × 1-CH_3), 1.77 (s, 6H, 2 ×

3-CH_3), 6.88 (m, 1H, 7-H), 7.09 (m, 3H, 4-/5-/6-H), 7.25 (quasi-s, 5H, α-phenyl) ppm, assigned through comparison with **42a**; for ^{13}C NMR, see ref [S8].

2-{Bis[*p*-(bromomethyl)phenyl]methylidene}-1,1,3,3-tetramethylindane (43): This unwanted side-product was isolated from the brominative deoxygenation of a sample of ketone **38a** that was contaminated by the tertiary alcohol **39a**. After separation from the desired bromoalkene **42a** through chromatography on silicagel (60 Å, 63–200 μm) with low-boiling petroleum ether/Et_2O (60:1), the almost pure fraction of **43** had a mp of 161–163 °C; ^{1}H NMR ($CDCl_3$, 400 MHz) δ 1.28 (s, 12H, 2 $\times$ 1-/3-CH_3), 4.46 (s, 4H, 2 $\times$ CH_2Br), 7.08 (m, AA´-part of an AA´BB´ system, ca. 2H, 4-/7-H), 7.20 (m, BB´-part, ca. 2H, 5-/6-H), 7.29 (quasi-s, 8H, 4 $\times$ *m*-H and 4 $\times$ *o*-H) ppm; ^{13}C NMR ($CDCl_3$, 100.6 MHz) δ 31.8 (2 $\times$ 1-/3-CH_3), 33.4 (2 $\times$ CH_2Br), 48.5 (C-1/-3), 122.1 (C-4/-7), 127.0 (C-5/-6), 128.6 (4 $\times$ C-*m*), 129.8 (4 $\times$ C-*o*), 135.7 (2 $\times$ C-*p*), 137.7 (C-α), 143.8 (2 $\times$ C-*ipso*), 149.8 (C-3a/-7a), 156.9 (C-2) ppm, assigned through comparison with benzyl bromide and tetraphenylethene.

3. Alternative generations and the behavior of the Cl,K-carbenoid 12

3.1. FBW ring expansion of 12

Potassium *tert*-butoxide (KO*t*-Bu), but not LiO*t*-Bu, is a sufficiently active base to deprotonate the monochloride **14** (Scheme S2) slowly at 70 °C. The resultant Cl,K-carbenoid **12** did not add to cyclohexene in THF as the solvent and was not trapped (at least not irreversibly) by di-*tert*-butyl ketone (*t*-$Bu_2C{=}O$) in cyclohexane. In both of these solvents, **12** expanded its five-membered ring to generate the hitherto unknown cycloalkyne **S6** in analogy with the earlier [S9] examples involving unsaturated Br,K- and Cl,Li-carbenoids. A run in heptane as the solvent (50 hours at 90 °C) provided evidence for the unknown enolate **S7** through quenching with solid CO_2, which furnished the β-ketoacid **S9** along with the known [S10] ketone **S8**. The chiral constitution of **S9** followed from its ^{1}H NMR spectrum in $CDCl_3$ which exhibited four nonequivalent methyl groups (δ_H = 1.29, 1.49, 1.55, and 1.61 ppm) and for the center of chirality a one-proton signal (δ_H = 3.98 ppm). The thermal lability of **S9** prevented its isolation and further characterization: The complete decarboxylation in $CDCl_3$ solution within four days at rt afforded the ketone **S8**, whose constitution [S10] established the ring expansion of carbenoid **12**. Because *tert*-butyl ethers deriving from **12** or **S6** were never detected, it remains unknown whether the enolate **S7** arose through hydration of **S6** by adventitious moisture or through a base-induced decay of a *tert*-butyl ether derived from **S6**.

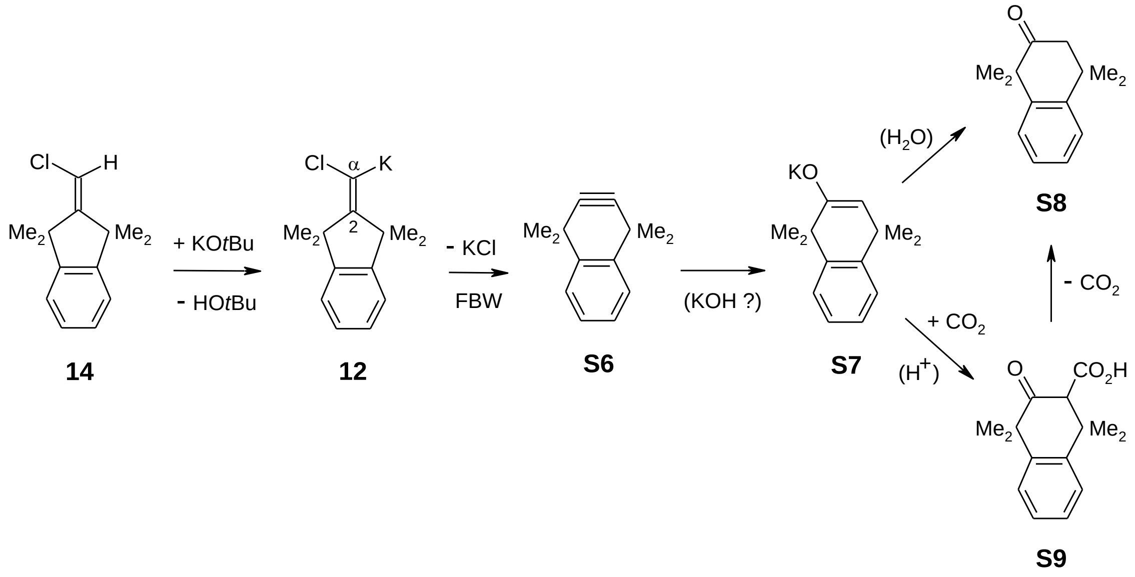

Scheme S2: FBW ring expansion of the Cl,K-carbenoid **12**.

1,1,4,4-Tetramethyl-2-tetralone (S8): A dry NMR tube (5 mm) was charged with the monochloride **14** (110 mg, 0.50 mmol), KO*t*-Bu (166 mg, 1.47 mmol), anhydrous THF (0.8 mL), and cyclohexene (0.2 mL). The tightly stoppered tube was heated at 70 °C for ca. 40 hours, emptied into aqueous hydrochloric acid (2 M, 10 mL), and shaken with Et_2O (3 × 10 mL). The combined Et_2O layers were washed with distilled water until neutral, dried over $MgSO_4$, and concentrated. After distillation at 75–90 °C (bath temp.)/0.001 mbar, some contaminations were removed through column chromatography on silica gel with low-boiling petroleum ether, whereupon the ketone **S8** was eluted from the column with Et_2O (yield 27%) and washed with cold pentane: mp 68–71 °C (ref [S10]: 75 °C); ^{1}H NMR ($CDCl_3$, 200 MHz) δ 1.31 and 1.46 (2 s, 2 × 6H, 2 × 1-/3-H), 2.65 (s, 2H, CH_2), 7.20–7.40 (m, 4H, aromatic protons) ppm.

3.2. The S_NV reaction of benzyl potassium (S11) with carbenoid 12

The easily prepared (Scheme S3) and purified [S11] benzyl potassium (**S11**) guides the dichloroalkene **6** into and through an efficient carbenoid chain reaction: **S11** acts not only as a chlorine acceptor in step 1 and as a nucleophile in step 2; it is also consumed by the coproduct $PhCH_2Cl$ of step 1 (giving dibenzyl), by the primary chain

product **S13** to produce the allene **S14**, and by deprotonating **S14** to afford **S15** which was recognized through carboxylation that generated the acid **S16**.

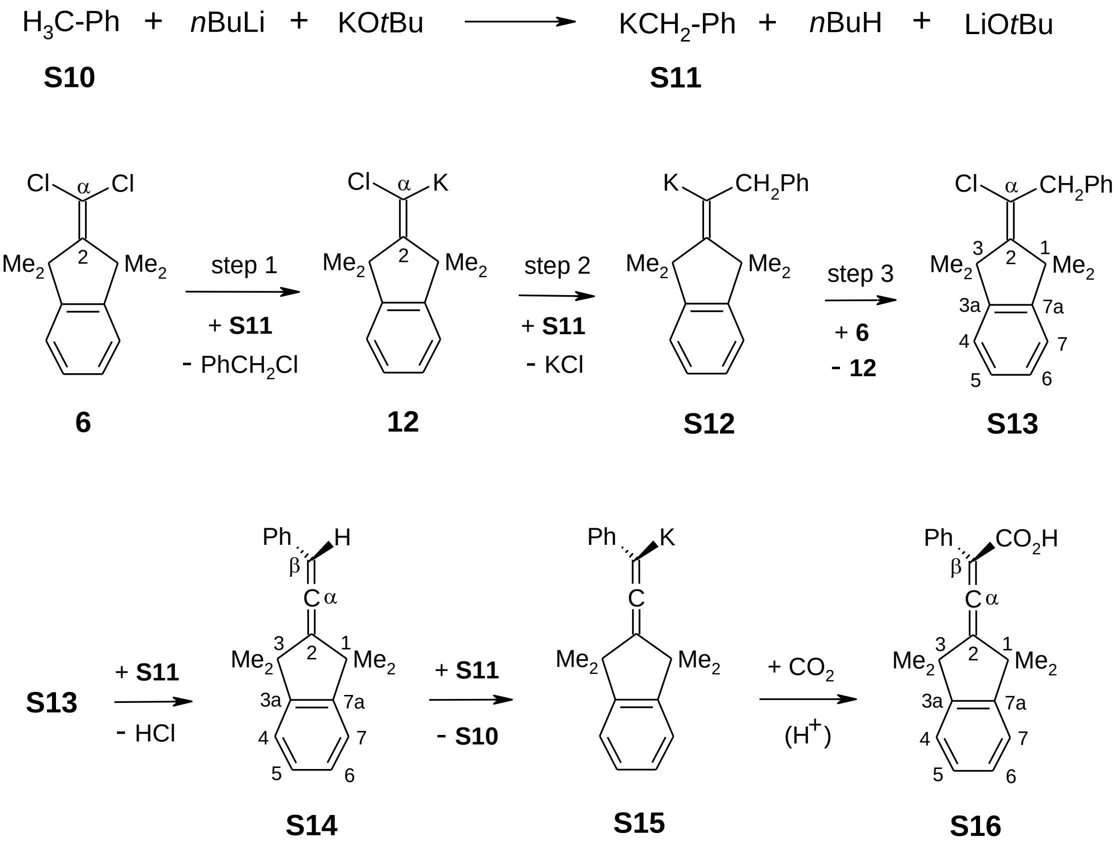

Scheme S3: Carbenoid chain S_NV of the Cl,K-carbenoid **12**.

2-Phenyl-3-(1,1,3,3-tetramethyl-2-ylidene)propenoic acid (S16): The solid dichloroalkene **6** (153 mg, 0.60 mmol) was added to an ice-cooled, red solution of $PhCH_2K$ (**S11**, max. 3.0 mmol) [S11] in anhydrous THF (2.0 mL) under argon gas cover. The instantaneously blackened mixture was stirred at rt for 30 min, then poured onto solid CO_2, warmed up, and dissolved in aqueous NaOH and Et_2O. The aqueous layer was washed with Et_2O, acidified with conc. hydrochloric acid, and extracted with Et_2O which afforded the crude acid **S16** together with phenylacetic acid. Crystallization from CCl_4 yielded colorless **S16** (11%), mp 230–231 °C after recrystallization (CCl_4); ^{1}H NMR ($CDCl_3$, 400 MHz) δ 1.52 and 1.56 (2 s, 2 × 6H, 2 × 1-/3-CH_3), 7.18 (m, 2H, 4-/7-H), 7.27 (m, 3H, *p*-H and 5-/6-H), 7.33 (tm, ^3J = 7.7 Hz, 2H, 2 × *m*-H), 7.64 (dm,

^{3}J = 7.7 Hz, 2H, 2 × *o*-H), 10.46 (broad s, CO_2H) ppm, assigned through comparison with compound **S9** in ref [S12]; ^{13}C NMR ($CDCl_3$,100.6 MHz) δ 30.89 and 31.43 (2 × 1-/3-CH_3), 49.03 (C-1/-3), 107.35 (C-β), 122.44 (C-4/-7), 127.62 (C-5/-6 and C-*p*), 127.99 (2 × C-*o*), 128.30 (2 × C-*m*), 129.30 (C-2), 132.80 (C-*ipso*), 148.15 (C-3a/-7a), 170.53 (CO_2H), 207.08 (C-α) ppm, assigned through comparisons with compound **S9** in ref [S12], with phenylallene, and with phenylacetic acid.

The product of workup through protolysis instead of CO_2 quenching contained **S13**, **S14**, and dibenzyl. After distillation at 90–190 °C (bath temp.)/0.1 mbar, fractional crystallizations from low-boiling petroleum ether and from methanol furnished enriched samples of **S13** and **S14**, respectively.

S13: Identified through the ^{37}Cl/^{35}Cl mass spectral intensity ratio of 1:3 and ^{1}H NMR comparison with compound **4f** in ref [S12]. ^{1}H NMR (CCl_4, 80 MHz) δ 1.55 (s, 6H, 2 × 1-CH_3), 1.68 (s, 6H, 2 × 3-CH_3), 4.00 (s, 2H, CH_2Cl), 7.21 (quasi-s, 9H, all aromatic protons) ppm; EIMS *m*/*z* (%): 312.2 and 310.2 (ca. 1:3, 8%, [M^+]), 297.2 and 295.2 (31% + 94%).

S14: Identified through a weak allene vibration in the IR (KBr) at ν: 1949 cm^{-1}; ^{1}H NMR (CCl_4, 80 MHz) δ 1.44 and 1.49 (2 s, 2 × 6H, 2 × 1-/3-CH_3), 6.39 (s, 1H, β-H), 7.14 (quasi-s, 9H, all aromatic protons) ppm.

3.3. No ring expansion during deprotonation and S_NV of monochloride 14 by the potassium amide KHMDS

It remained to demonstrate that a less reactive potassium nucleophile than **S11**, provided that it is more soluble than KO*t*-Bu, is also able to perform the S_NV reaction before the ring expansion becomes perceptible. We observed by in situ ^{1}H NMR spectroscopy how mixtures of $KN(SiMe_3)_2$ (KHMDS, potassium 1,1,1,3,3,3-hexamethyldisilazide) and $HN(SiM_3)_2$ (HMDS, 1,1,1,3,3,3-hexamethyldisilazane) [S13]

deprotonated the monochloride **14** (Scheme S4). The consumption of **14** required six days at rt in toluene with *t*-BuOMe (6:5 vol/vol) but only three hours in THF (where LiHMDS did not react over days at rt). In *t*-BuOMe as the solvent, **14** (0.07 M) was consumed by KHMDS (0.13 M) and HMDS (0.14 M) at rt with a first half-reaction time of ca. seven hours. In all cases, the enamine **S18** was the main (and the only identified) product; ring expansion generating the cycloalkyne **S6** did not take place. Final evidence for the S_NV reaction was obtained through hydrolysis of the crude material containing the enamine **S18** which furnished the known [S14] aldehyde **S19** as the only descendant.

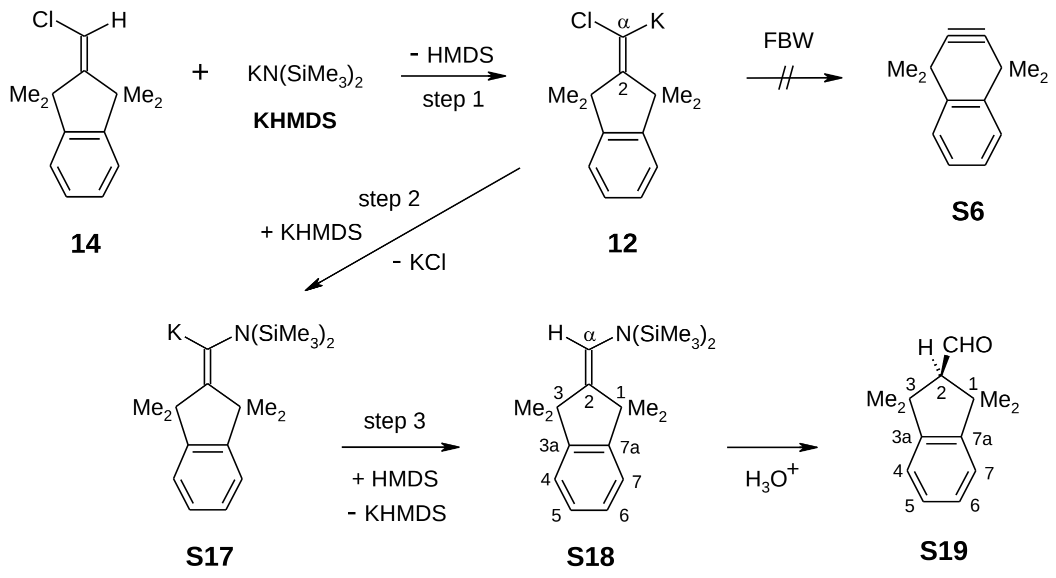

Scheme S4: No FBW ring expansion of **12** with $KN(SiMe_3)_2$.

$KN(SiMe_3)_2$ as a base and nucleophile: A weighed, dry NMR tube (5 mm) was charged with potassium hydride in mineral oil (49 mg) and pentane (0.3 mL). The suspension was whirled up through gentle shaking, and the turbid supernatant was withdrawn by syringe from the heavy precipitate of KH. After twofold repetition of such leaching, the residual pentane was removed in a soft stream of dry argon gas emanating from a long pipette for at least 5–15 seconds, leaving dry KH powder (29

mg, ≤0.72 mmol) which was suspended in anhydrous -*t*BuOMe (0.65 mL) and treated with HMDS (0.040 mL, 0.20 mmol) and [D_{12}]cyclohexane (0.040 mL). As expected [S15,S16] for such an unactivated specimen of KH, the proton transfer from HMDS (evolution of H_2) took five days at rt for a partial (ca. 50%) formation of KHMDS (0.11 mmol). The monochloride **14** (11 mg, 0.05 mmol) was added and observed to be consumed over two days at rt. The final mixture was dissolved in Et_2O/water (vivid but short evolution of H_2), and the Et_2O layer was washed with distilled water until neutral, dried over Na_2SO_4, and gently evaporated to afford the slightly contaminated enamine **S18** (15 mg, 94%): ^{1}H NMR of **S18** ($CDCl_3$, 200 MHz) δ 0.17 (s, 18 H, 2 × $SiMe_3$), 1.39 (s, 6H, 2 × 3-CH_3), 1.53 (s, 6H, 2 × 1-CH_3), 5.99 (s, 1H, α-H), 7.12–7.24 (m, 4H, 4-/5-/6-/7-H) ppm, assigned through comparison with the analogous α-$OSiMe_3$ derivative [S17].

Upon treatment of this $CDCl_3$ solution with aqueous hydrochloric acid (2 M, three drops), the enamine **S18** vanished within 150 min, and the known [S14] aldehyde **S19** emerged: ^{1}H NMR ($CDCl_3$, 200 MHz) δ 1.43 and 1.44 (2 s, 2 × 6H, 2 × 1-/3-CH_3), 2.54 (d, ^3J = 4.0 Hz, 1H, 2-H), 7.17 (AA´ part of an AA´BB´ system, 2H, 4-/7-H), 7.25 (BB´ part, 2H, 5-/6-H), 10.03 (d, ^3J = 4.0 Hz, 1H, CHO) ppm.

4. References used in the Supporting Information

S1. Knorr, R.; Lattke, E.; Räpple, E. *Liebigs Ann. Chem.* **1980**, 1207–1215, compound **4** therein.

S2. Dawe, R. D.; Molinski, T. F.; Turner, J. V. *Tetrahedron Lett.* **1984**, *25*, 2061–2064.

S3. Knorr, R.; Freudenreich, J.; von Roman, T.; Mehlstäubl, J.; Böhrer, P. *Tetrahedron* **1993**, *49*, 8837–8854.

S4. We thank Mrs. Petra Böhrer and Dr. J. Mehlstäubl for preparing this compound.

S5. We thank Dr. T. P. Hoang for synthesizing this compound.

S6. Knorr, R.; von Roman, T.; Freudenreich, J.; Hoang, T. P.; Mehlstäubl, J.; Böhrer, P.; Stephenson, D. S.; Huber, H.; Schubert, B. *Magn. Reson. Chem.* **1993**, *31*, 557–565, compound **23** in Table 3 therein.

S7. von Roman, U.; Ruhdorfer, J.; Knorr, R. *Synthesis* **1993**, 985–992, method A1 therein and quoted literature, compound **11j** therein.

S8. Compound **25** in Table 3 of ref [S6].

S9. Knorr, R. *Chem. Rev.* **2004**, *104*, 3795–3849, p 3824 therein.

S10. Bruson, H. A.; Grant, F. W.; Bobko, E. *J. Am. Chem. Soc.* **1958**, *80*, 3633–3636.

S11. Knorr, R. *Chem. Eur. J.* **2012**, *18*, 7506–7515, pp 7510 and 7513 with refs [34] and [35] therein.

S12. Knorr, R.; Pires, C.; Behringer, C.; Menke, T.; Freudenreich, J.; Rossmann, E. C.; Böhrer, P. *J. Am. Chem. Soc.* **2006**, *128*, 14845–14853.

S13. For δ_H values of KHMDS and HMDS, see the Supporting Information of ref [S11].

S14. Compound **41** in ref [S3].

S15. Soderquist, J. A.; Rivera, I. *Tetrahedron Lett.* **1988**, *29*, 3195–3196.

S16. Hubbard, J. L. *Tetrahedron Lett.* **1988**, *29*, 3197–3200.

S17. Compound **14** in ref [S6].
